# Enhancing Rehabilitation Practices: Over- and Undertreatment in a German Outpatient Sexual Offender Program: Monitoring Degrees of Treatment Based on Standardized Risk Levels

**DOI:** 10.5964/sotrap.12893

**Published:** 2025-12-19

**Authors:** Markus Dietl, Sharon Schumann, Markus G. Feil

**Affiliations:** 1 Diakonie Munich, Munich, Germany; 2 inforensik, Munich, Germany; Simon Fraser University, Burnaby, Canada; University Medical Center Mainz, Mainz, Germany

**Keywords:** standardized risk levels, sexual offender treatment programs, degree of over/under treatment, social therapy, prison-based and outpatient treatment needs, German rehabilitation policy

## Abstract

**Background:**

Effective reintegration into the community requires tailored treatment programs that address criminogenic needs. Ultimately, these efforts are essential to maintaining public safety.

**Objective:**

This study aimed to evaluate whether our outpatient program met the criminogenic needs of participants.

**Method:**

Between 2008 and 2023, treatment needs of a sample of 955 male sexual offenders were assessed using the Static-99 tool and standardized risk levels.

**Results:**

A significant 63.5% of the offenders exhibited treatment needs. Among the total population, 56% were undertreated, 14% overtreated, and 30% adequately treated. Therapy in sociotherapeutic facilities had a significant influence on over- and undertreatment.

**Conclusion:**

The degrees of treatment based on standardized risk levels are proving to be very valuable in forensic outpatient care, underscoring an urgent requirement for more individualized treatment strategies. Sociotherapeutic interventions play a key role in reducing undertreatment.

## Abstract (plain language summary)

**Background:**

Quality problems in treatment mainly include over- and undertreatment. Undertreatment happens when patients do not receive the necessary treatment, while overtreatment involves giving excessive or unnecessary treatment, which can be harmful and represents an unnecessary consumption of limited resources. The essential goal is adequate treatment, which should be timely and beneficial. Our study uses standardized risk levels to provide insights into recidivism risk and recommend adequate treatment duration based on treatment needs. Our main goal was to ensure the correct amount of treatment for sexual offenders.

**Why was this study done?:**

A German outpatient sexual offender program monitored treatment degrees based on standardized risk levels to prevent over- and undertreatment, enhancing rehabilitation practices.

**What did the researchers do and find?:**

Data from the past 15 years was perused, focusing on sexual offenders attending our programme. Regrettably, a majority were found to be undertreated. Special attention was given to patients undergoing treatment in sociotherapeutic facilities (STFs), which are specialized departments within prisons where psychotherapeutic interventions take place. These facilities often significantly extend treatment times, which has a positive impact on the need for treatment.

**What do these findings mean?:**

This observation highlights the alarming trend that high-risk patients often withdraw from treatment early. A promising answer lies in combining imprisonment with STF to ensure completion of the program, a cornerstone of the rehabilitation journey. This urges the need for full support and specialized programs.

## Highlights

- Degrees of treatment help tailor sexual offender programs, focusing on recidivism risk to balance treatment intensity.
- Over 50% of patients in our outpatient program are receiving less care than required for their risk levels.
- Utilizing standardized risk levels to guide treatment planning ensures adequate treatment for all offenders, enhancing rehabilitation outcomes and minimizing inefficiencies.
- The unmet treatment needs of high-risk inmates, resulting from undertreatment in prison-based sociotherapeutic facilities, place a burden on the limited resources of outpatient programs.

Effective treatment for sexual offenders requires evidence-based interventions that focus on criminogenic needs to reduce recidivism (Hanson et al., 2009). Balancing treatment intensity is crucial to avoid over- or undertreatment, ensuring better outcomes (Lohr & Schroeder, 1990; Lowenkamp & Latessa, 2002). Therefore, the program's outpatient practice increasingly uses evidence-based practices in risk assessment and tailors treatment to offenders individual risk factors and various backgrounds (Drawbridge et al., 2019; Hogan & Olver, 2023).

## Challenges in Implementing Effective Treatment

One of the challenges in the treatment of sexual offenders is the variability in recidivism; Rettenberger et al. (2015) report an overall recidivism rate of 6% within five years in Austria, varying between rapists (4%) and child molesters (8%), while Lussier et al. (2023) report Canadian rates around 10%. Longitudinal data shows recidivism increases over time, with 10-year rates 1.5 times higher than 5-year rates (Lee & Hanson, 2021), underscoring the need for long-term monitoring. In addition, risk assessment tools have inconsistent agreement rates ranging from 23% to 71%, adding complexity to treatment management (Jung et al., 2013). Risk-Need-Responsivity (RNR) model provides a framework to address these challenges (Hanson et al., 2017b).

## Risk-Need-Responsivity (RNR) Model

The RNR model is fundamental to sexual offender treatment, emphasizing interventions tailored to individual risk factor (Andrews et al., 1990; Bonta & Andrews, 2007). The model is guided by three principles:

- **Risk Principle:** Treatment intensity should match the offender's risk level; high-risk offenders require more intensive interventions, low-risk offenders less (Gendreau, 1996).
- **Need Principle:** Focusing on criminogenic needs, like pro-criminal attitudes, helps reduce recidivism (Hanson et al., 2009).
- **Responsivity Principle:** Treatment should be tailored to the offender’s characteristics, including cognitive abilities, motivation, and personal circumstances (Birgden, 2004).

## Criminogenic Needs

Standardized risk levels based on RNR principles focus on the criminal risk associated with antisocial behavior (Bonta & Andrews, 2007). The Central Eight risk factors include both static and dynamic elements, such as criminal history, procriminal attitudes, antisocial personality traits, and substance abuse (Bonta & Andrews, 2016). While dynamic factors, like emotional regulation and social skills, may change with treatment, static factors, such as criminal history do not (Eher et al., 2021; Olver et al., 2007). Criminogenic needs refer to dynamic risk factors that can be modified through treatment. Sexual offender rehabilitation must address both dynamic and static factors in outpatient and prison-based programs (Prenzler et al., 2023).

## Standardized Risk Levels

From 2014 to 2016, experts in Washington developed a five-level sexual offender risk assessment system (Hanson et al., 2017b; Justice Center, 2014), which assigns individuals to one of five levels based on risk scores (see Table 1).

**Table 1:** Examining Outcomes Using the Standardized Risk Levels

Level	Risk	Static-99 Score*	Prognosis	Criminogenic Needs	5 Years Recidivism** (A)	5 Years Recidivism*** (C)
I	Low	0	Excellent	None or Few	1%	1%
II	Moderate-Low	1-2	Very good	Few	2%	3%
III	Moderate	3-4	Good	Multiple	5%	8%
IVA	Moderate-High	5-6	Improvement	Multiple	14%	15%
IVB	High	> 7	Improvement	Multiple	31%	53%

*Note.* A = Austria; C = Canada. Source based on: Chassin and Galvin (1998).

*Eher et al. (2019). **Eher et al. (2021). ***Hanson et al. (2017a).

These risk levels help allocate treatment resources, ensuring high-risk offenders receive intensive treatment (Guéridon & Suhling, 2020) and low-risk offenders avoid unnecessary interventions, improving overall treatment efficiency (Viljoen et al., 2018). Tools such as the Static-99 and VRS-SO tailor strategies to reduce recidivism (Eher et al., 2019; Olver et al., 2007). The VRS-SO includes dynamic factors but we use Static-99 for completeness in the sample, focusing on static factors for long-term risk assessment and resource allocation (Eher et al., 2019).

### Monitoring Degrees of Treatment in Rehabilitation

It is essential to track the effects of treatment in the forensic setting. Treatment degrees include adequate, over- and undertreatment (Chassin & Galvin, 1998), help guide interventions. Hanson et al. (2017b) recommend 100-200 hours for Level III, 200-300 hours for Level IVA, 300+ hours for Level IVB and recommend specific treatment time based on criminogenic needs (Kroner & Hanson, 2023). Adequate treatment is set at 2 hours for Level I and 15 hours for Level II. These thresholds in Table 2 optimize rehabilitation.

**Table 2:** Definition of the Degrees of Treatment Based on the Standardized Risk Levels

Degrees of Treatment	Definition	I	II	III	IVA	IVB
Undertreatment	Patients do not receive services that could be beneficial.	–	–	< 100 h	< 200 h	< 300 h
Overtreatment	The services exceed what is necessary.	> 2 h	> 15 h	> 200 h	> 300 h	–
Adequate Treatment	Services that have a positive impact.	< 2 h	< 15 h	100 h – 200 h	200 h – 300 h	> 300 h

*Note.* h = hour. The degrees of treatment categorize the adequacy of therapeutic interventions provided based on standardized risk levels, influencing rehabilitation outcomes. Source: Hanson et al. (2017b), Chassin and Galvin (1998). For detailed methodologies or specific contexts, refer to the respective studies.

### Tailored Application in Sexual Offender Treatment

Forensic officers need to customize interventions based on best practice approaches. Matching therapy with offender risk profiles prevents undertreatment of high-risk offenders and improves resource use (de Leon, 2014; Doblytė, 2020). Applying the concept of degrees of treatment ensures that interventions are in line with offender characteristics (Dietl & Korczak, 2011). This supports evidence-based practices aimed at reducing recidivism by addressing cognitive abilities, motivation, and personal circumstances (Feil et al., 2024a).

## Treatment Approaches for Sexual Offenders in Germany

In Germany, treatment is structured around criminal law. Offenders found to be not fully responsible are treated in psychiatric hospitals, while others are treated in sociotherapeutic facilities (STFs) (Lösel et al., 2020). Rigorous therapy quality monitoring with clear benchmarks and outcome measures is essential to improve treatment effectiveness. However, meta-analyses on reducing recidivism rates have shown mixed results. Individualized therapy may be needed (Schmucker & Lösel, 2015). Some programs report positive outcomes (Gannon et al., 2019), but continued evaluation is necessary. Studies in Germany and Switzerland show significant recidivism reductions in outpatient settings (Franke et al., 2021; Seewald et al., 2018).

### Gold Standard Treatment Protocols

The RNR and Good Lives Model (GLM) are essential frameworks for effective treatment planning (Bonta & Andrews, 2007; Ward & Gannon, 2006; Yates, 2009). These models incorporate psychological assessments to tailor interventions (Schwarze et al., 2018). In German outpatient programs, RNR is integrated into 71.9% of cases, while GLM is used in 62.5%, adjusting treatment intensity based on recidivism risk (Gregório Hertz et al., 2019).

## Outpatient Sexual Offender Programs

The study examines an outpatient sexual offender treatment program in Munich and southern Bavaria, aiming to reduce recidivism and support social reintegration (Carl & Schmucker, 2017) by assessing risk, improving impulse control, and promoting prosocial relationships (Schwarze et al., 2018).

### Risk Assessment in Outpatient Programs

Risk assessment plays a central role in treatment (Olver & Stockdale, 2020), using tools like Static-99, Stable-2007 (Eher et al., 2021), and VRS-SO (Olver et al., 2007) to evaluate static and dynamic factors. A case manager monitors initial risk assessments and serves as a contact person to external parties, while the therapist focuses on treatment. In Munich, these roles are separated, but this is not standard practice across Germany.

### Interdisciplinary Therapy Approach

An interdisciplinary team of psychologists, psychotherapists, and social workers develops an individualized treatment plan (Schwarze et al., 2018). The goal is to address criminal behavior develop a sense of responsibility, and promote socially appropriate behavior. Cognitive-behavioral therapy is the most common approach (46%), followed by systemic (17%) and psychodynamic therapy (16%). Medication is used alongside psychotherapy in 11% of cases (Gregório Hertz et al., 2019; Turner et al., 2013).

### Outpatient Group Therapy Details

Group therapies typically last 60-90 minutes and involves approximately eight participants (Schulte Ostermann et al., 2021). Psychoeducational sessions are used to motivate high-risk offenders with low engagement in treatment (Feil & Furjanic, 2019). Mentalization-Based Therapy (MBT) is effective for offenders with Antisocial Personality Disorder, and improves emotional regulation and social skills (Flaaten et al., 2024). Interactive psycho educational groups are effective for child pornography offenders (Rimer, 2021) and schematherapy has been shown to change criminal behavior help change maladaptive patterns associated with (Schulte Ostermann et al., 2021). Participants discharged from STFs are prioritized for these programs.

## Rehabilitation in Sociotherapeutic Facilities

STFs provide psychotherapeutic and social interventions aimed at addressing offenders' criminogenic needs (Kury & Fenn, 1977).

### Rationale for Investigating STFs

A significant number of sexual offenders are released without receiving prison-based treatment, leading to gaps in access (Carl & Lösel, 2021b). Investigating STFs may enhance treatment availability and effectiveness, particularly for offenders who did not receive appropriate interventions. Understanding operations can inform policy and resource allocation, ensuring more equitable treatment options (Bussmann & Richter, 2013; Carter & Whitworth, 2015), ultimately improving rehabilitation outcomes (Carl et al., 2019; Carl & Lösel, 2021a).

### Focus of STF Programs

In Bavaria, STFs typically target low to moderate-risk offenders. In some facilities, up to 93% of low-risk individuals are treated (Carl et al., 2019), even though standardized assessments indicate no need for treatment (Hanson et al., 2017b). High-risk offenders benefit most from specialized treatment (Hanson et al., 2009; Schmucker & Lösel, 2015). The discrepancy between patients' risk profiles and their actual treatment illustrates the complexity of treatment needs, comparable to treating low fever with intensive care while high fever patients are ignored. STF programs employ evidence-based therapies like cognitive-behavioral techniques to address psychological factors driving criminal behavior (Rocha & Valença, 2023). These interventions reduce recidivism and improve rehabilitation outcomes (Schmucker & Lösel, 2015).

## Objective of the Study

The purpose of this study is to analyze the distribution of treatment levels (defined as adequate, over-, and undertreatment) across standardized risk levels in our sexual offender program. Specifically, we investigate whether high-risk offenders receive inadequate treatment while low-risk offenders may be overtreated. High-risk offenders typically require more intensive interventions (Mailloux et al., 2003), and existing research highlights the importance of aligning treatment intensity with an offender's risk level to reduce recidivism (Bonta et al., 2000).

Furthermore, this study evaluates how over- and undertreatment impact rehabilitation outcomes for inmates in STFs. Research suggests that limited resources may lead to undertreatment for high-risk offenders and overtreatment for low-risk offenders. In a worst-case scenario, placing low-risk offenders in high-intensity programs may increase their risk of recidivism (Lowenkamp & Latessa, 2002). The central question, therefore, is whether there is a significant relationship between treatment degrees and standardized risk levels, underscoring the importance of tailored interventions for effective rehabilitation.

## Method

### Study Location and Participants

A retrospective cohort study was conducted in a forensic outpatient clinic in Munich.

**Inclusion Criteria:** Male sexual offenders assessed using the Static-99 tool during their initial consultation from 2008 to July 2023.

**Exclusion Criteria:** Subjects were excluded due to non-participation in the first consultation, inapplicability of the Static-99 tool, dropout or discontinuation of services, excessive distance from the therapy location and duplicate entries. Additionally Records of individuals who relapsed and later returned to the outpatient program were consolidated to prevent data duplication.

**Assessment Tool:** The Static-99 tool, translated into German by Eher et al. (2012), was used to assess and classify risk levels. This tool took into account factors such as age, previous criminal record, and victim details (Harris et al., 2003; Kube & Banse, 2020).

### Calculation of Treatment Time

In our outpatient program, treatment time is calculated based on time spent across several phases, including initial consultation during the risk assessment stage, socio-therapeutic discussions, and both individual and group therapy sessions during the pre-treatment and intervention phases. This breakdown provides a comprehensive view of the time allocated to all treatment phases.

In STFs, calculating treatment time must account for practical limitations. Our estimate is approximately 150 effective treatment hours per inmate, based on individual and group therapy sessions. In comparison, Carl et al. (2019) estimate 288 hours: 48 hours of individual therapy and 240 hours of group therapy per year. However, according to Bussmann and Richter (2013), nearly 30% of participants discontinue individual therapy, and 15% discontinue group therapy. Adjusting for practical issues such as illness and facility operations, our revised estimate of 150 hours reflects more realistic conditions.

### Statistical Methods

Demographic and treatment data were collected from risk assessments and internal documentation, including information on STF experience, group therapy, and other treatment factors.

#### Hypothesis Formation

Researchers hypothesized that treatment degrees affect standardized risk levels in sexual offenders. Our hypotheses were as follows:

**Primary Hypothesis:** Higher-risk levels are linked to higher undertreatment and lower overtreatment chances in outpatient settings.

**Secondary Hypothesis:** Receiving forensic therapy at STF has a significant impact on the degrees of treatment.

#### Data Analysis Strategy

The data was analyzed to understand its impact on treatment outcomes. The approach involved calculating demographic and treatment-related statistics.

##### Metric Variables

Age, Static-99 Score, and treatment time were reported as mean (*M*) and standard deviation (*SD*) and were used as continuous variables. When data did not follow a normal distribution, the Kruskal-Wallis and Mann-Whitney tests were used to compare treatment groups.

##### Normality Assessment

To select the appropriate statistical test, the Shapiro-Wilk test was used to check whether the quantitative data were normally distributed. None of the variables met normality assumptions. Treatment time and Static-99 Score was noticeably skewed to the right of the histogram, although the age was somewhat bell-shaped, but still did not qualify as normal.

##### Selection of Statistical Tests

Initially, we tried ANOVA and the *t*-test for age, but the results reflected the results of the Kruskal-Wallis and Mann-Whitney *U* tests. To ensure careful and robust results, we focused only on nonparametric testing. This approach keeps the analysis valid despite the lack of data health. The use of nonparametric tests such as the Kruskal-Wallis test or the Mann-Whitney *U* test is consistent with best practices for non-normally distributed data and ensures the reliability of the results.

##### Categorical Variables

Chi-square (χ^2^) tests were employed to examine categorical variables, including Risk Assessment and Intervention Stage, Treatment Indication, Group Therapy, Type of Offense, and standardized risk levels. This approach facilitated our understanding of their distribution across various treatment categories, thereby enhancing the robustness of our analysis.

#### Logistic Regression Analysis

To evaluate the impact of previous experience with STFs on the degrees of treatment and treatment needs (III-IVB), we performed a logistic regression analysis. The examination concentrated on the following aspects:

##### Outcome Variables

Logistic regression forecasts results by analyzing various variables. It estimates the probability of certain outcomes contingent upon defined variables. This research emphasizes key outcome variables. Firstly, treatment needs (III-IVB) are analyzed, representing the number of participants needing treatment based on standardized risk levels. The analysis links these needs to treatment time (Table 2). Next, treatment degrees were examined, with adequate treatment coded as 1 (sufficient) and 0 (not). Overtreatment was coded as 1 (excessive) and 0 (not). Undertreatment was coded as 1 (insufficient) and 0 (not), ensuring clarity in outcome analysis.

##### Independent Variable

The STF experience was coded as 1 or 0 to investigate its association with treatment needs (III-IVB) and treatment degrees.

The logistic regression analysis results are presented in Table 5, including detailed coefficient estimates (*B*), standard errors (*SE*), *p*-values, odds ratios (Exp(*B*)), and 95% confidence intervals (CI) for each outcome variable. This comprehensive presentation illustrates the influence of STF experience on the degree and treatment and treatment needs (standardized risk level III-IVB).

##### Justification for the Use of p-Values

*p*-values were crucial for determining statistical significance and ensuring reliable analysis. Each *p*-value tested hypotheses and relationships, with many findings having *p*-values below .05, supporting hypotheses on STF experience's impact on treatment effectiveness. The consistent use of *p*-values confirmed the validity of the assessment.

## Results

### Demographics and Sample Characteristics

#### Distribution of Sexual Offenders

This study analyzed 955 male sexual offenders using the Static-99 tool, evaluating treatment needs (III-IVB) and STF experience. Results indicated significant differences in treatment adequacy based on STF experience: 17.7% of offenders had STF experience, while 82.3% did not. Overall, adequate treatment was received by 30.5% of offenders, overtreatment was recorded for 13.8%, and undertreatment occurred in 55.7% of cases (see Table 3a).

**Table 3a:** Descriptive Statistics of Demographic and Risk Assessment Data by Treatment Degree and Sociotherapeutic Facility Experience

Variable	Degrees of Treatment						Sociotherapeutic Facility			
	Total *N* = 955	Adequate Treatment *n* = 291	Over-treatment *n* = 132	Under-treatment *n* = 532	Test Statistics	*p*	STF Experience *n* = 160	STF Inexp. *n* = 795	Test Statistics	*p*
	*n* (%)	*n* (%)	*n* (%)	*n* (%)	χ^2^ (*df*)		*n* (%)	*n* (%)	χ^2^ or *U* (*df*)	
*N* (%)	955 (100)	291 (30.5)	132 (13.8)	532 (55.7)	254.8	< .001	160 (17.7)	795 (82.3)	422.2	< .001
Age *(M, SD)*	48.8 (15.6)	52.7 (15.2)	54.6 (12.7)	45.3 (15.6)	45.2 (2)	< .001	47.0 (13.0)	49.2 (16.1)	*U* = 67847(1)	.182
Static-99 Score *(M, SD)*	3.4 (2.0)	1.9 (1.3)	1.4 (1.0)	4.7 (1.4)	566.4 (2)	< .001	3.8 (2.1)	3.3 (2.0)	7.2 (1)	.007
Treatment needs (III-IVB)	606 (100.0)	64 (10.6)	10 (1.7)	532 (87.8)	699.9 (2)	< .001	112 (18.5)	494 (81.5)	240.8 (1)	< .001
Risk Assessment Stage	555 (100)	196 (35.3)	53 (9.5)	306 (55.2)	174.0 (2)	< .001	44	511	392.9 (1)	< .001
Intervention Stage	400 (100)	95 (23.7)	79 (19.8)	226 (56.5)	97.6 (2)	< .001	116	284	70.6 (1)	< .001

*Note.* STF = Sociotherapeutic Facility; Inexp = Inexperience. The "STF Experience" variable refers to participants treated in prison-based sociotherapeutic facilities, while "STF Inexp." refers to those treated only in outpatient settings.

#### Disparities in Treatment Time

Adequately treated sexual offenders received a median of 4 hours of treatment, while overtreated received 95 hours and undertreated received 10 hours. Offenders with STF experience received 162 hours, compared to 6 hours without STF experience, highlighting inconsistent treatment allocation. This variance affects rehabilitation outcomes, emphasizing the need for standardized treatment based on risk and needs (see Table 3b).

**Table 3b:** Treatment Information

Variable	Degrees of Treatment						Sociotherapeutic Facility			
	Total *N* = 955	Adequate Treatment *n* = 291	Over-treatment *n* = 132	Under-treatment *n* = 532	Test Statistics	*p*	STF Experience *n* = 160	STF Inexp. *n* = 795	Test Statistics	*p*
	*n* (%)	*n* (%)	*n* (%)	*n* (%)	χ^2^ (*df*)		*n* (%)	*n* (%)	χ^2^ or *U* (*df*)	
Treatment Time (h)	11.4	4.1	95.0	9.8	115.7 (2)	< .001	162	6	*U* = 678 (1)	< .001
*(Mdn, Q0.25, Q0.75)*	[2.6-71.3]	[1.7-150.0]	[25.8-161.0]	[2.5-33.1]			[151-195]	[2-18]		
Treatment h *(M, SD)*	50.7 (80.0)	52.1 (80.1)	105.2 (83.1)	33.9 (54.4)	115.7 (2)	< .001	179.3 (39.2)	18.7 (31.3)	*U* = 678 (1)	< .001
Treatment Indication	234 (100.0)	42 (17.9)	62 (26.5)	130 (55.6)	53.2 (4)	< .001	45 (19.2)	189 (80.8)	88.6 (1)	< .001
STF Experience	160 (100.0)	55 (34.4)	57 (35.2)	48 (30.0)	0.8 (2)	.658	–	–		–
Group Therapy	133 (100.0)	18 (13.5)	36 (27.1)	79 (59.4)	44.3 (2)	< .001	25 (18.8)	108 (81.2)	51.8 (1)	< .001

*Note.* h = hours; *Mdn* = Median; Q = Quantile; *SD* = Standard Deviation; STF = Sociotherapeutic Facility; Inexp = Inexperience. "STF Experience" refers to participants treated in prison-based STFs, while "STF Inexp" indicates those treated only in outpatient settings. "Treatment Indication" denotes the need for specific interventions based on risk assessments.

#### Offenses

The study of 955 sexual offenders found significant undertreatment rates for various crime types. Exhibitionism had no offenders with STF experience, while 41.6% of sexual abuse cases with known victims, 83.3% with unknown victims, 61.3% of sexual assault, and 53.1% of rape with known victims were undertreated (see Table 3c).

**Table 3c:** Distribution of Offenses

Variable	Degrees of Treatment						Sociotherapeutic Facility			
	Total *N* = 955	Adequate Treatment *n* = 291	Over-treatment *n* = 132	Under-treatment *n* = 532	Test Statistics	*p*	STF Experience *n* = 160	STF Inexp. *n* = 795	Test Statistics	*p*
	*n* (%)	*n* (%)	*n* (%)	*n* (%)	χ^2^ (*df*)		*n* (%)	*n* (%)	χ^2^ or *U* (*df*)	
Exhibitionism	44 (100.0)	3 (6.8)	1 (2.3)	40 (90.9)	76.6 (2)	< .001	–	44 (100)	–	–
Child Pornography	30 (100.0)	7 (23.4)	4 (13.3)	19 (63.3)	8.3 (2)	.016	6 (20.0)	24 (80.0)	10.8 (1)	.001
Sexual Abuse Known	411 (100)	157 (38.2)	83 (20.2)	171 (41.6)	42.7 (2)	< .001	76 (18.5)	335 (81.5)	163.5 (1)	< .001
Sexual Abuse Unknown	66 (100.0)	8 (12.2)	3 (4.5)	55 (83.3)	87.1 (2)	< .001	8 (12.1)	58 (87.9)	37.9 (1)	< .001
Sexual Coercion	137 (100.0)	48 (35.0)	5 (3.6)	84 (61.3)	55.7 (2)	< .001	12 (8.8)	125 (91.2)	93.2 (1)	< .001
Rape Known	175 (100.0)	56 (32.0)	26 (14.9)	93 (53.1)	28.4 (2)	< .001	37 (21.1)	138 (78.9)	58.3 (1)	< .001
Rape Unknown	60 (100.0)	10 (16.7)	3 (5.0)	47 (78.3)	69.7 (2)	< .001	19 (31.7)	41 (68.3)	8.1 (1)	.007

### Risk Levels and Degrees of Treatment

#### Distribution of Sexual Offenders Across Risk Levels

The distribution of sexual offenders across standardized risk levels (I, II, III, IVA, IVB) and the adequacy of their treatment were examined (see Table 2). Significant differences in treatment outcomes emerged, particularly concerning adequate, overtreatment, and undertreatment. Analysis revealed considerable disparities in the degrees of treatment provided across different risk levels, underscoring substantial variability in the treatment approaches based on offenders’ assessed risk. These findings are detailed in Table 4. Notably, 64% of the participants were distributed across risk levels II and III, and only 12.4% in levels I and IVB. High-risk offenders (III and IVB) were more likely to receive inadequate treatment, with 81.4% and 98.2% undertreated, respectively.

**Table 4:** Distribution of Sexual Offenders by Standardized Risk Levels and Treatment Degrees

Level	Cases (*N* = 953)	Adequate Treatment (*n* = 290)	Overtreatment (*n* = 132)	Undertreatment (*n* = 531)	Test Statistics	*p*
	*N*	*n* (%)	*n* (%)	*n* (%)	χ^2^ (*df*)	
I	61 (6.4)	32 (52.5)	29 (47.5)	0.0	0.2 (1)	.70
II	287 (30.1)	194 (67.6)	93 (32.4)	0.0	35.5 (1)	< .001
III	323 (33.9)	51 (15.8)	9 (2.8)	263 (81.4)	344.4 (2)	< .001
IVA	225 (23.6)	12 (5.3)	1 (0.4)	212 (94.3)	376.2 (2)	< .001
IVB	57 (6.0)	1 (1.8)	0 0.0	56 (98.2)	108.1 (2)	< .001

*Note. N* represents the total number of sexual offenders in each risk level category. The percentages represent the proportion of cases within each risk level out of the total sample (*N* = 953). The percentages indicate also the proportion of cases within each risk level, along with percentages of adequate, over, and under treatment. It also includes χ^2^ test statistics noting significant differences in treatment degrees across risk levels with p-values less than .001.

#### Validation of Study Hypothesis

The results support the hypothesis that higher-risk individuals receive inadequate treatment, emphasizing the need for targeted interventions.

### Effects of STF Experience

The study examines how participating in the STF programs affects treatment degrees for sexual offenders, using logistic regression analysis. The table shows odds ratios (Exp(*B*)) and 95% confidence intervals (CI) for different treatment adequacy outcomes based on STF participation: Treatment needs (III-IVB), adequate, overtreatment, and undertreatment.

#### Effects of STF Participation on Treatment Needs

Participating in STF programs is only marginally associated with treatment needs (III-IVB), with an odds ratio of 1.42 (95% CI [0.99, 2.05]). The logistic regression model explains a significant amount of variance in treatment needs (III-IVB) outcome (Cox & Snell *R*^2^ = 0.38). However, the model's accuracy in predicting this outcome is moderate, at 0.64.

#### Impact of STF Experience on Undertreatment and Overtreatment

For undertreatment, STF participation significantly reduces the odds (Exp(*B*) = 0.28, 95% CI [0.19, 0.40], indicating a protective effect. Conversely, participation in the STF program significantly increases the odds of overtreatment (Exp(*B*) = 5.31, 95% CI [3.56-7.94]).

#### STF Participation and Adequate Treatment

There is no statistically significant relationship between STF participation and adequate treatment (Exp(*B*) = 1.24, 95% CI [0.87, 1.78]). These findings highlight the complex impact of STF experience on treatment outcomes for sexual offenders. (see Table 5).

**Table 5:** Effect of STF Experience: Results of Logistic Regression Analysis

Outcome Variable	Measure *N* = 160	*B*	*SE*	*p*	Exp(*B*)	95% CI	Cases (*n*)	Accuracy
Treatment needs (III-IVB)	STF	0.35	0.19	.06	1.42	0.99 - 2.05	606	0.63
Undertreatment	STF	-128.96	0.19	< .001	0.28	0.19 - 0.40	423	0.56
Overtreatment	STF	16.70	0.20	< .001	5.31	3.56 - 7.94	132	0.14
Adequate Treatment	STF	0.22	0.18	.24	1.24	0.87 - 1.78	291	0.30

*Note.* *B* = Coefficient estimate; *SE* = Standard error; Exp(*B*) = Odds ratio; 95% CI = 95% Confidence Interval; STF = Sociotherapeutic Facility. The table shows the results of logistic regression analysis examining the effect of STF experience on treatment outcomes, with the "Accuracy" column representing correct predictions for each outcome.

## Discussion

Our study aimed to evaluate whether our forensic outpatient program provided need-based treatment for sexual offenders between 2008-2023. We assessed how the STF program affects treatment degrees.

### Summary

Our findings highlight the degrees of treatment as a meaningful outcome in forensic therapy research. Standardized risk levels effectively quantify both over- and undertreatment. Insufficient treatment was more common, while excessive treatment occurred less frequently. These results underscore the need for individualized treatment strategies, beginning during incarceration and continuing post-release.

### Detailed Findings

#### Treatment Disparities by Risk Level

Significant disparities were found between treatment received and risk levels. Higher-risk offenders (III, IVA, IVB) were often undertreated, indicating a need for targeted interventions. Additionally, those completing the STF program tended to receive overtreatment, a pattern corroborated by prior research (Carl et al., 2019; Lowenkamp & Latessa, 2002). While expanding the STF program may help address undertreatment, attention must be paid to the risk of overtreatment, particularly for lower-risk offenders (Bonta et al., 2000; Mailloux et al., 2003). Statistically significant differences (*p* < .05) were observed in the relationship between STF completion and treatment degrees, indicating the need for more individualized treatment approaches.

### Interpretation of Findings

Effective rehabilitation is influenced by individual, institutional, and political factors. At the individual level, tailored therapy plans that address specific risks and criminogenic needs are essential. Hoffman and Pearson (2009) highlight the need to balance over- and undertreatment to achieve optimal rehabilitation outcomes. Institutionally, maintaining treatment quality is crucial, as both over- and undertreatment can diminish effectiveness (Chassin & Galvin, 1998). Institutions should prioritize strategies that align treatment intensity with risk levels to minimize disparities (Wertz et al., 2023). At the political level, policymakers must adopt evidence-based strategies to improve treatment for high-risk individuals, avoiding the practice of focusing on "easier" cases ("creaming") while neglecting more challenging ones ("parking") (Carl et al., 2019; Carter & Whitworth, 2015).

### Implications

This study emphasizes key considerations for treating high-risk individuals in forensic therapy. The results highlight the need for evidence-based practices. This discussion delves into practical implications for improving treatment outcomes and advocates for informed policy adjustments to better address the complex needs of sexual offenders.

#### Recommendations for Enhanced Treatment Delivery

We recommend following prescribed treatment time to reduce risks associated with untreated sexual offenders (Hanson et al., 2017b). However, completing more than 300 hours of outpatient treatment is often difficult. This treatment time is the equivalent of nearly five years of regular psychotherapy and represents a significant challenge for court-mandated offenders (Feil et al., 2024b). Therefore, it would be more realistic to achieve these treatment goals in an inpatient setting where such external barriers are minimized, and incentives (such as financial compensation or privileges) can be offered.

#### Group Therapy in Forensic Settings

Group therapy in forensic settings can enhance social skills and reduce recidivism (Lösel & Schmucker, 2005), but challenges exist in meeting diverse criminogenic needs of sexual offenders. Combining group therapy with personalized assessment protocols can improve treatment outcomes and address needs more effectively (Feil & Furjanic, 2019; Feil et al., 2024b).

#### Methodological Insights

The 15-year retrospective analysis of longitudinal data from a forensic outpatient program highlights critical patterns of over- and undertreatment. These findings suggest that incorporating real-world data into treatment planning can guide more efficient resource allocation (Gandjour & Lauterbach, 2004).

#### Enhancing Treatment Engagement Through STF

STF methods significantly reduced early dropouts among high-risk individuals, showing its role in maintaining engagement, particularly with high-risk offenders (Kury & Fenn, 1977). STF integration into programs is advised to boost engagement and lower recidivism (Guéridon & Suhling, 2020). In long-term settings, remotivating dropouts is easier compared to outpatient settings.

### Further Questions

Our study emphasizes the need for further research into the criminogenic treatment needs of sexual offenders. While we examined over- and undertreatment, the connection between initial offenses and treatment requirements is still uncertain. Future studies should explore how specific offenses impact treatment needs and investigate the causes of overtreatment, particularly in cases where high-risk individuals may be misidentified. Understanding criminogenic needs and risk factors that contribute to criminal behavior could help develop more effective treatment strategies (Kroner & Hanson, 2023).

### Limitations

#### Study-Specific Limitations

Our study has methodological limitations that may impact conclusions.

##### Unequal Sample Distribution

A major limitation is the uneven distribution of risk levels. Most of our participants fall into level III, differing from the level II norm reported by Eher et al. (2012), raising concerns about the applicability of our results to the larger German-speaking population. Furthermore, only 7.1% of participants were in levels IVA and IVB, limiting the study’s conclusions for higher-risk individuals.

##### Transferability Issues in Use of Static-99

The Static-99 tool, while commonly used in our program (Rettenberger & Craig, 2020), has limitations in assessing cases like child pornography, where victims are less identifiable (Eher et al., 2019). It also offers limited insight into criminal attitudes or employment, critical factors in preventing recidivism (Olver et al., 2018).

##### Limited Analysis Options Due to VRS-SO Exclusion

Although we initially aimed to include VRS-SO data, it was excluded due to its smaller sample size (344 compared to 955 for Static-99) and the belief that it wouldn't provide significant additional insights. Focusing on Static-99 ensured our study’s relevance. Future studies could explore VRS-SO’s role in assessing dynamic risk factors (Feil et al., 2024b).

##### STF Time Based on Assumptions

The study analyzed file reviews but lacked detailed documentation on the actual therapy time provided in STF programs. As a result, we relied on Carl et al.’s (2019) estimate of 150 hours for completed treatment, which may oversimplify the complexities of treatment delivery. This assumption-based estimation could affect the accuracy of our findings, highlighting the need for more precise recording of therapy time in future research.

#### General Limitations

Several limitations affect the interpretation and generalizability of our findings.

##### Incomplete Data on Recidivism Rates

The lack of comprehensive data on recidivism rates among sexual offenders presents a significant limitation, impeding effective policymaking (Etzler et al., 2020). Without reliable data, it is challenging for policymakers to develop evidence-based strategies aimed at preventing repeat offenses, resulting in inefficient resource allocation.

##### Variations in Recidivism Data

Comparisons between recidivism risks in Austria (Eher et al., 2021) and Canada (Hanson et al., 2017a) show hardly any differences. Eher reported a gradual increase in recidivism, from 1% at stage I to 31% at IVB. Hanson's numbers were higher but comparable. Recidivism at the highest level differed the most, at 31% versus 53% for IVB.

##### Limitations in Model Accuracy

Our models moderately predict treatment needs and undertreatment but demonstrate limited accuracy in predicting overtreatment and adequate treatment, highlighting the complexity of forecasting forensic outcomes. This limitation may stem from data variability, potential biases, and intricate relationships between variables. To improve model accuracy, more sophisticated modeling techniques and expanded datasets are necessary. Future research should incorporate diverse data sources and advanced methodologies to enhance the precision of treatment planning and inform evidence-based forensic interventions. The study provides valuable insights into treatment stratification, but its limitations require cautious interpretation and emphasize the need for continued research to improve forensic therapy practices.

## Data Availability

Due to legal and ethical restrictions related to data protection and the sensitive nature of forensic patient information, the data that support the findings of this study are not publicly available. Data may be shared with qualified researchers upon reasonable request to the corresponding author, subject to approval by the relevant ethics committee and institution.
